# A case of early epileptic encephalopathy caused by new mutation at W218C in *KCNQ2* and review literature

**DOI:** 10.1016/j.bbrep.2025.102008

**Published:** 2025-04-29

**Authors:** Juhua Yang, Yuping Huang, Zhijun Chen, Jiaheng Peng, Kangyu Li, Lijuan Huang, Jie Yang, Chunhui Yang

**Affiliations:** a Department of Neonatology, Zhongshan Boai Hospital, Zhongshan, 528400, China; b Department of Neonatology, Nanfang Hospital, Southern Medical University, Guangzhou, 510450, China; c Department of Pediatrics, Zhongshan Boai Hospital, Zhongshan, 528400, China

**Keywords:** Newborn, Early epileptic encephalopathy, *KCNQ2*, Genotype-phenotype

## Abstract

Early-onset epileptic encephalopathy (EOEE) is mainly characterized by early refractory epileptic seizures in infants with progressive brain dysfunction, accompanied by complex causes (such as perinatal brain injury, structural brain malformations and genetic metabolic diseases). Early identification and etiological treatment are critical. It has been reported that mutations in Potassium Voltage-Gated Channel Subfamily Q Member 2 (*KCNQ2*) can result in EOEE. This study analyzed the genetic defects and clinical phenotypes of a newborn with early epileptic encephalopathy. Whole exome gene detection identified a novel heterozygous point mutation p. W218C in *KCNQ2.* The pathogenic variant was located in the protein's S4S5 connection region and was identified as a harmful mutation by silico tools. The child's clinical phenotype finally manifested as West syndrome during the follow-up. The mentioned variation may lead to severe clinical manifestations and poor neurological prognosis. Whole exome gene detection provides clinicians with more information on neonatal epileptic encephalopathy.

## Highlights

• Our findings identify a novel missense mutation at the W218C locus within the *KCNQ2* gene, which is associated with severe clinical manifestations and unfavorable neurological outcomes.
• These results provide valuable insights for clinical genetic counseling and offer novel perspectives for future investigations into the pathogenesis of this disorder.

## 1 Introduction

Genetic factors have been proven to be involved in the pathogenesis of early-onset epileptic encephalopathy (EOEE) by generating and pruning synapses, differentiation and migration of neurons, and ion transport. Additionally, about 265 genes are related to EOEE [1]. Potassium Voltage-Gated Channel Subfamily Q Member 2 (*KCNQ2*) gene encodes neuronal voltage-gated potassium channels. Its variation can lead to epilepsy, mainly distributed in the S4 voltage receptor, C terminal, membrane area, pore area (S5S6), with the most common missense mutation [2]. It has also been reported that its variation in the S4, pore area and C-terminal special function has been linked to early epileptic encephalopathy (including West or Ohtahara syndrome) [3,4]. As a result, identifying *KCNQ2* pathogenic variants and their phenotypes is essential for genetic counseling and patient management.

This study reports a case of a child with early epileptic encephalopathy. A novel heterozygous mutation, p. W218C, was identified in the *KCNQ2* gene through Whole exome gene detection. According to silico tools prediction and structural analysis, the new pathogenic variant destroy the structure of the KCNQ2 protein, thus affecting its function. Unlike previous reports, this pathogenic variant is located in the S4S5 junction region of the KCNQ2 protein rather than the special region of S4, pore region, and C-terminus. Moreover, in previous reports, the T217 mutation in this region led to benign familial neonatal seizures, not early epileptic encephalopathy. Additionally, there are few cases of the *KCNQ2* pathogenic variants leading to the development of early epileptic encephalopathy into West syndrome. Therefore, the genotype and phenotype database of *KCNQ2*-EOEE was enriched in this study, which provided a reference for clinical genetic counseling and new insights into clinical research of the disease.

## 2 Method

### 2.1 Patient and clinical data collection

Detailed clinical data of a neonatal convulsion patient hospitalized in Zhongshan Boai Hospital in 2023 were collected.

### 2.2 Gene detection and biological software analysis

Peripheral blood from the patient and his parents were collected for whole exome sequencing (WES) and copy number variation (CNV) detection analyses after the consent of the family. Whole exome high-throughput sequencing technology was used to capture exons,and the mutation site detection system was used for comparative analysis with reference to the human genome 19 (GRCh37/hg19) database.

### 2.3 Prediction and conservation analysis using silico tools

The biological online software PROVEAN (http://provean.jcvi.org/index.php) and Polymorphism Phenotyping v2 (PolyPhen-2, http://genetics.bwh.harvard.edu/pph2/) were used to predict the deleteriousness of the mutation. Conservatism analysis was performed using an online comparison by CLUSTALW (https://www.genome.jp/tools-bin/clustalw). The Combined Annotation Dependent Depletion (CADD, https://cadd.gs.washington.edu/snv) tool was used to score the deleteriousness of the mutation. A CADD score greater than 15 was considered to be potentially harmful.

### 2.4 Molecular modeling of wild-type and mutant KCNQ2 proteins and the interaction between phosphatidylinositol 4,5-diphosphate (PI(4,5)P2; PIP2) and KCNQ2

Molecular modeling of wild-type and mutant KCNQ2 proteins: AlphaFold3 software was used to predict the structure of KCNQ2 protein, and PyMOL was used to visualize and analyze the structure of the protein model. The amino acids in the sequences of wild-type and mutant proteins were arranged in the alignment of PyMOL software to highlight their similarities and differences. The two protein models exhibited a high degree of similarity in structure, as evidenced by the alignment of 872 residues. The similarity between the models is further supported by the high comparison score (4595.000). The wild-type and mutant protein sequences of the matrix contrast field were compared using Matrix:EBLOSUM62 in ClustalOmega, and the results revealed that they are the same at almost all positions (99.8 %) and have no gaps, indicating a high level of identity and coverage.

Previous studies suggest that the formation of KCNQ2 tetramer may be regulated by endogenous molecules such as calmodulin (CaM) and phosphatidylinositol 4,5-diphosphate (PI(4,5) P2; PIP2). PIP2 is a necessary cofactor for KCNQ channel activation, which stabilizes the open state of the channel by polar or electrostatic interaction with the S4S5 connection domain and adjacent regions [5]. Consequently,the effect of 218 site variations on the interaction process was analyzed in this study. The PIP2 ligand structure was downloaded from the small molecule website (PubChem: https://pubchem.ncbi.nlm.nih.gov/#query=GlycerophosphOino-sitol%204 %2C5-bisphosphate). The CB-Dock2 server (https://cadd.labshare.cn/cb-dock2/in-dex.php) was used to perform molecular docking of CaM–KCNQ2 tetramer (Alphafold3 predicted generation, structure score:wild-type ipTM = 0.68, pTM = 0.7; mutant:ipTM = 0.67,pTM = 0.69) and PIP2 based on computer automation, and the docking results were visualized using Discovery studio and Ligplus.

### 2.5 Document retrieval method

The China National Knowledge(CNKI) and Wanfang databases were searched for newborn and Potassium Voltage-Gated Channel Subfamily Q Member 2 OR KCNQ2. The biomedical literature database (PubMed) was also searched for infant AND/OR neonate and Potassium Voltage-Gated Channel Subfamily Q Member 2 OR KCNQ2 for literature review.

## 3 Results

### 3.1 Case information of the children

**Clinical manifestations:** One day after birth, a 12-day-old girl was admitted to the hospital after experiencing intermittent convulsions for 11 days. The infant kept twitching while in the hospital, which presented as either head to one side, binocular gazing, double upper limbs, and right lower rigidity, without any jitter; there was no blood oxygen decline, no fever, no rash, poor self-sucking milk, oral feeding.

**Pregnant and childbirth history:** The child is the second child, with a gestational age of 38^+3^ weeks, no signs of premature rupture of membranes and intrauterine distress. She was born at the Zhongshan Chenxinghai Hospital of Integrated Traditional Chinese and Western Medicine at 16:12 on March 28, 2023. There was no history of resuscitation, amniotic fluid, and placenta abnormalities; the umbilical cord remained around the neck for 1 week; the Apgar score of 1, 5, and 10 min was 10 points, and the birth weight was 2600g. The history of pregnant women includes gestational diabetes, poor blood glucose control during pregnancy, hepatitis B three positive, maternal blood type 'A' type Rh (+), and parents are Guangdong people.

**Fig. 1:**
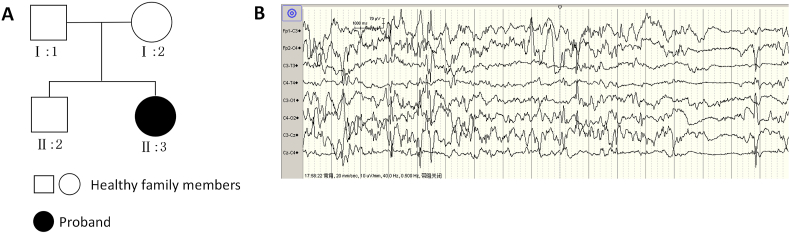
A: The parents and brother of the child had no epilepsy; B: The EEG of the child illustrating multifocal and multi-volume spike slow.

**Family history:** Denial of family history of epilepsy, and family situation of the proband(Fig. 1A).

**Physical examination for admission:** Physical examination revealed T36.8 °C, P139 times/min, R46 times/min, BP71/37 mmHg,and TCSO_2_ 94 %. Full-term infants had the appearance, a clear mind, good response, no convulsions, no paleness, no yellow staining, soft anterior fontanelle, equal round and large pupil on both sides, no shortness of breath, no three concave signs, soft anterior fontanelle, no resistance to the neck, thick respiratory sounds in both lungs, no dry and wet rales, uniform heart rhythm, strong heart sounds, and no murmur in the precordium. The abdomen was not swollen, the liver was not under the ribs, the bowel sounds were normal, the muscle tension of the limbs was normal, and the original reflex was extracted.

**Auxiliary examination:** There was no abnormality in infection index, electrolyte, lactic acid, blood ammonia, blood glucose, and cerebrospinal fluid examination. During the out-of-hospital imaging consultation, the brain computed tomography and magnetic resonance imaging(MRI) plain scans revealed no abnormalities. Video electroencephalograph monitoring(EEG) illustrated moderate abnormal neonatal EEG, unclear sleep cycle, many multifocal sharp waves, sharp slow waves, spike waves, and spike slow waves. Multiple EEG monitoring indicated seizures and multifocal and multi-volume spike slow waves (Fig. 1B). Re-examination of EEG monitoring revealed an upper boundary of 40–70 μV, a lower boundary of > –10 μV, and a lack of sleep-wake cycle. The original EEG demonstrated that the whole-conduction spike-slow wave was not synchronized and continuously issued. In conclusion, severe abnormality and peak arrhythmia were observed.

**Treatment process:** After admission, phenobarbital was administered to stop convulsions, and a high-dose vitamin B6 was supplemented for 5 days. The children still experienced convulsions. EEG monitoring identified many multifocal sharp waves, sharp slow waves, spike waves, and spike slow waves, so levetiracetam was used as an anti-epilepsy medication and gradually increased. Finally, even after receiving 5 mg/kg/d of phenobarbital and 42 mg/kg/d of levetiracetam, the child continued to have convulsions about 2–3 times/day.

**Prognosis:** The children were diagnosed with West syndrome, with dystonia and psychomotor retardation,and experienced nodding and hugging seizures at around 8 months of age, multifocal sharp wave, head, temporal area, and widespread irregular high amplitude slow wave, and several isolated epileptic seizures. Adrenocorticotropic Hormone(ACTH,2 IU/kg∗d) combined with magnesium sulfate, oral clobazam, valproic acid, and oxcarbapine were administeres to the patient, the nodding and embracing seizures improved, but mental retardation persisted.

### 3.2 Gene detection, prediction, and conservation analysis of the mutation

**Fig. 2:**
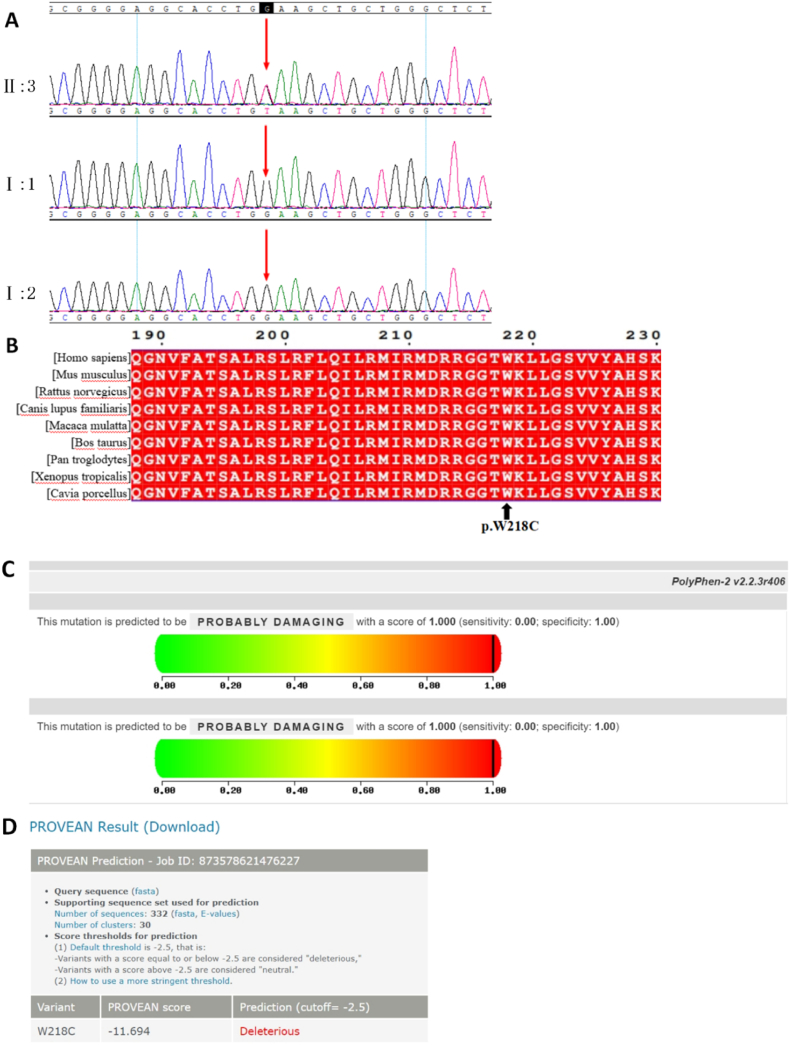
Gene detection and silico tools analysis results; A: Whole exome sequencing revealed a KCNQ2 mutation (c.654G > T, p. W218C) in the proband (II:3). Sanger verification and qPCR verified normal genotypes in the parents (I: 1, I: 2) of the child. The patient had a spontaneous de novo heterozygous missense mutation; B: The variation is conservative in many species; C and D: PROVEAN and PolyPhen-2 predicted that the mutation was harmful.

CNV detection did not reveal any significant variation. Whole-exome sequencing revealed a KCNQ2 mutation, c.654G > T(p.Trp218Cys,p.W218C). Verification by Sanger sequencing and quantitative polymerase chain reaction (qPCR) identified normal genotypes in the child's parents. Therefore,the patient had a spontaneous de novo heterozygous missense mutation (Fig. 2A). The variation was conserved in many species (Fig. 2B). It was predicted to be harmful by the biological software PROVEAN and PolyPhen-2 (Fig. 2C and D). With a CADD score of 37,the variation may be harmful.

#### 3.2.1 The structural changes introduced by the amino acid substitution (p.W218C)

**Fig. 3:**
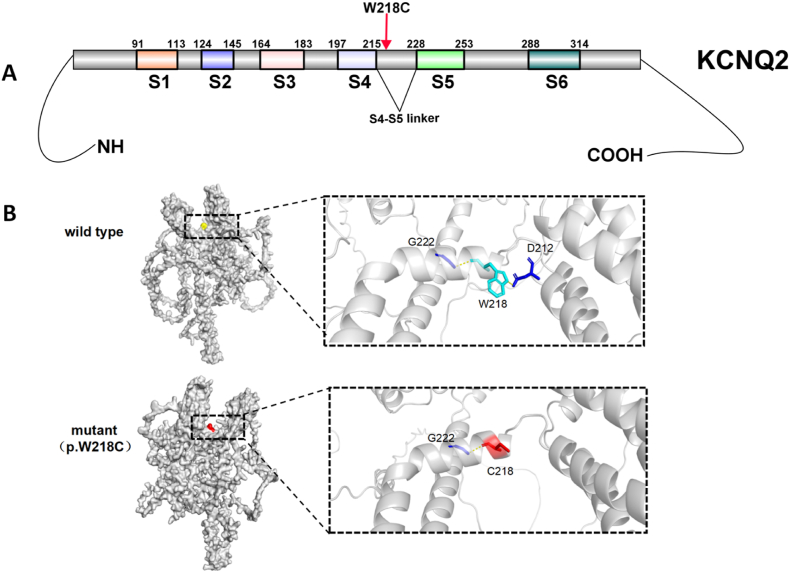
Molecular modeling of KCNQ2 protein; A: p. W218C in KCNQ2 was located in the S4S5 junction region of KCNQ2 protein (IBS 1.0.3); B: PyMOL software was used to construct the model before and after KCNQ2 mutation.

The p. W218C in *KCNQ2* mutation was located in the S4S5 junction region of KCNQ2 protein (Fig. 3A). The PyMOL software was used to construct the model before and after the KCNQ2 mutation. The blue is the wild-type (wt) amino acid, the red is the mutant amino acid, and the hydrogen bond is represented by the yellow dotted line (Fig. 3B). This point mutation cleaves amino acids at 218 and 212 positions, which may enhance the interaction with the amino acid at 222 position. Additionally, hydrogen bond cleavage occurs around the mutation site. This may change the local secondary structure and function of the protein.

#### 3.2.2 The change of interaction with PIP2 caused by amino acid substitution (p.W218C)

**Fig. 4:**
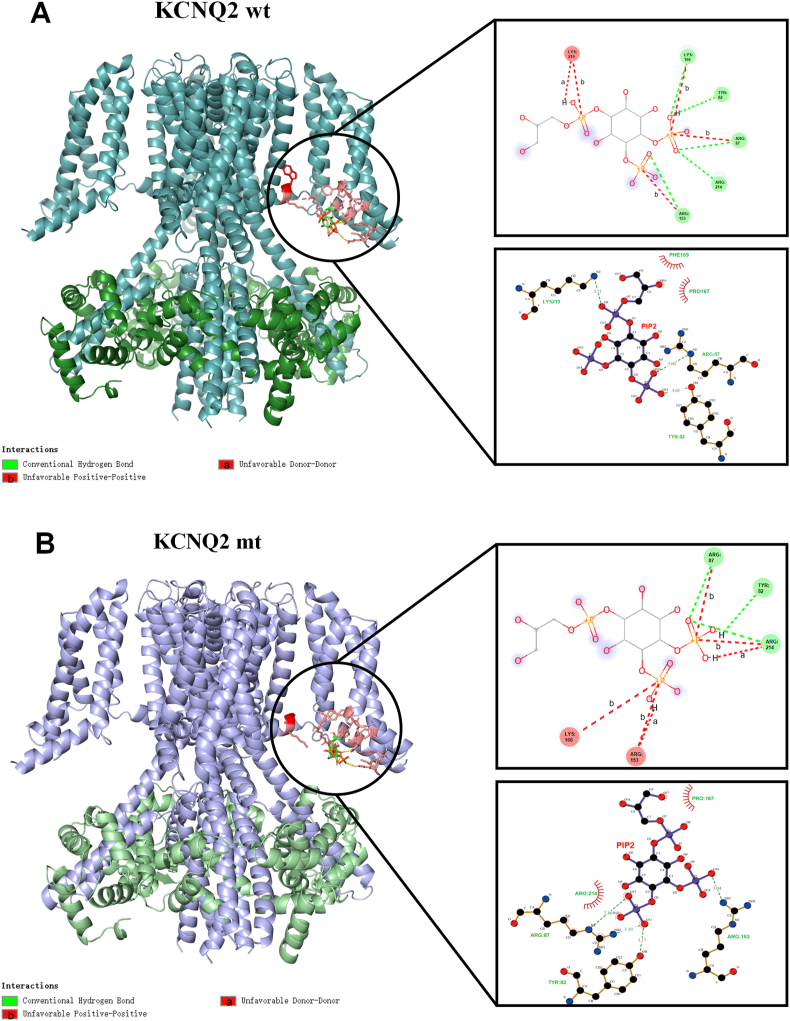
The interaction between PIP2 and KCNQ2; A: KCNQ2 wild type: Cyan represents KCNQ2, and green represents CaM.The circular frame highlights the specific situation of the docking part. The central part is the PIP2 ligand. All the pink residues represent the residues in 5A around the PIP2 ligand, including the red 218W.From top to bottom,on the left are the analysis results for Discovery Studio and Ligplus software, respectively.In combination, there is a hydrogen bond (stable binding),an unfavorable charge repulsion (may limit the binding direction), and an acceptor/donor conflict (the conformational adjustment is required to bind) between the 219 site and PIP2 in the wild type. B: KCNQ2 mutant: Purple represents KCNQ2,and light green represents CaM. The circular frame highlights the specific situation of the docking part. The central part is the PIP2 ligand. All pink residues represent the residues in 5A around the PIP2 ligand, including the 218C of the red display variant. From top to bottom,on the left are the analysis results for Discovery Studio and Ligplus software. In combination, the above interaction between the 219 site and PIP2 in the wild type completely disappeared, and the role of other residues was the same as before.

Based on the structural changes of the interaction between PIP2 and KCNQ2 (Fig. 4A and B), the potential mechanism was speculated to be as follows: (1) Spatial steric hindrance: the cysteine (C) side chain is short, resulting in local conformational changes so that PIP2 cannot be close to the 219 site. (2) Destruction of charge environment: The aromatic ring of tryptophan (W) may be involved in hydrophobic or π–π stacking, which weakens local charge complementarity and reduces the affinity of PIP2 after mutation. (3) Loss of dynamic stability: The mutation of tryptophan (W)- > cysteine (C) is located at the edge of the S4 domain. The mutation may increase the flexibility of the S4S5 connection domain, making it difficult to maintain the precise conformation required for PIP2 binding.

### 3.3 *KCNQ2* variations and clinical features based on literature review

The genotype and phenotype data published in the neonatal period are summarized (Table 1). *KCNQ2* pathogenic variants are distributed throughout the gene, resulting in different severity and missense mutations, which are the most common among these.

**Table 1:** Clinical and mutational profiles of published neonatal *KNCQ 2* deficiency cases.

*KCNQ2* variant type	Variation site	Protein region	Age of onset	Clinical manifestation	Electroencephalogram	Treatment	Reference
Missense mutation	p.Tyr127Asp	S2	6 h	Tonic seizure	Epileptiform discharges in multiple brain regions	Oxcarbazepine	17
	P.Val182Met	S3	1 d	Asymmetric rigidity with clonus	Multifocal spike (sharp) waves with a slow background rhythm	Phenobarbital, oxcarbazepine, levetiracetam, sodium valproate, topiramate, lamotrigine	15
	p.Arg210His	S4	1 d	Tonic asymmetric, apnea	Burst-suppression	Phenobarbital, phenytoin sodium	16
	p.Ala246Pro	S5	1 d	Tonic seizure	High degree of irregularity, partial burst suppression	Phenobarbital, levetiracetam, sodium valproate	18
	p.Thr274Met	H5	20 h	Generalized tonic seizures Multifocal clonic	Burst-suppression	Henobarbital, levetiracetam, topiramate	19
	p.Ala306Val	S6	2 d	Focal seizures	Burst-suppression	Phenobarbital, oxcarbazepine, levetiracetam	14
	p.Pro285Ser	Extracellular	0.5 h	Asymmetric rigidity	Bilateral asymmetric spikes with discontinuous low voltage	Phenobarbital, topiramate, sodium valproate	15
	p.Val 225Asp	Cytoplasmic	8 d	Myoclonus with torso rigidity	Burst-suppression	Sodium valproate, levetiracetam	15
	p.Arg333Trp	C terminal	5 d	Focal seizures	Multifocal, generalized epileptiform discharges	Phenobarbital, topiramate	14
	p.Arg553Gln	C terminal	1 d	focal seizures	multifocal, generalized epileptiform discharges	phenobarbital, levetiracetam	14
Samesense mutation	p.Leu129=(c.387G > A)	S2	4 d	Focal seizures	Nnormal	Oxcarbazepine	14
Nonsense or frameshift mutation	p.Asp681Thr fs∗249	C terminal	3 d	Focal seizures	Multifocal, generalized epileptiform discharges	Oxcarbazepine, levetiracetam	14
	p.Tyr284Ter	H5	3 d	Focal seizures	Multifocal, generalized epileptiform discharges	Phenobarbital, oxcarbazepine	14
Splice-site mutation	c.514+1G > A	S3	1 d	Pastic seizures of rigidity	Normal	Oxcarbazepine	14
Initiation codon mutation	c.1A > C	–	4 d	Pastic seizures of rigidity	Atypical outbreak-inhibition	Topiramate	17
Large fragment deletion	Chr20:61405051–62235975; 830.92 kb(KCNQ2, EEF1A2, CHRNA4)	–	3 d	Focal seizures	Normal	Oxcarbazepine, levetiracetam	14

## 4 Discussion

*KCNQ2* is a protein-coding gene widely expressed in the nervous system,and it combines with KCNQ3 to form a potassium channel [6]. The *KCNQ2* pathogenic variant is one of the most common causes of hereditary epilepsy in childhood, with an estimated incidence of 1 in 17000 during the first three postnatal years [7].

With the development of genetic technology, *KCNQ2* mutations leading to different severity of epilepsy phenotypes are being reported more frequently. The patient developed intractable seizures on the first day after birth. Genetic testing revealed p. W218C in the *KCNQ2* gene, leading to the diagnosis of early epileptic encephalopathy. The first case of early epileptic encephalopathy caused by the *KCNQ2* pathogenic variant (*KCNQ2*-EOEE) was reported by Dedek K et al., in 2003 [8]. *KCNQ2*-EOEE is characterized by multiple daily seizures, mainly tonic seizures, with associated focal motor and autonomic features from the first week of life. At the onset, EEG revealed sudden inhibition mode or multifocal epileptic activity, while early brain MRI identified a high density of basal ganglia; later, it demonstrated white matter or total volume loss. Epileptic seizures generally end between the ages of 9 months and 4 years of age. Moderate to severe developmental disorders may be exhibited [9]. The patient presented with multiple tonic-clonic or focal tonic seizures daily that were difficult to control with drugs. During the attack, EEG displayed multifocal multiple sharp spikes and slow waves, which was in line with the clinical characteristics of the disease. Unfortunately, at about 8 months of age, the patient developed a nodding and hugging spasm-like attack. According to the EEG, the interictal period was mainly characterized by multifocal sharp waves, spike waves, sharp slow waves scattered and clustered, and many bilateral posterior heads, temporal areas, and generalized irregular high-amplitude slow wave bursts. The patient was diagnosed with West syndrome, dystonia, and psychomotor retardation. The prognosis was poor. Few reports have previously documented the development of *KCNQ2*-EOEE into West syndrome [10].

Vitamin B6, phenobarbital, and levetiracetam were used for the treatment of the patient in the neonatal period, but the convulsions continued. When the gene was reported, the child was diagnosed with *KCNQ2*-EOEE,which later developed into West syndrome. The convulsions of the child improved when ACTH(2 IU/kg∗d) was administered in combination with magnesium sulfate, oral clobazone, valproic acid, and oxcarbazepine as an antiepileptic treatment, but mental retardation and poor prognosis continued. This is consistent with previous reports that *KCNQ2*-EOEE requires multiple drug treatments and has a poor neurological prognosis [11]. Therefore, genetic counseling and early treatment decision are vital. Some studies have recommended using sodium channel blockers (such as phenytoin sodium and carbamazepine) as a treatment for newborns with *KCNQ2*-EOEE, but the safety needs to be evaluated [12,13]. There are two reasons for this recommendation. First, sodium channel blockers prevent the movement of sodium ions through the channel during the propagation of action potentials, thereby blocking and preventing the development of epileptic seizures. Second, voltage-gated sodium channels and KCNQ potassium channels seem to be completely different voltage-gated ion channels, but they co-localize and bind to the key positions on the neuronal membrane; a structure-function approach explains the response of patients with potassium channel dysfunction to sodium channel blockers, indicating that regulating a channel may significantly affect the function of the channel complex [13]. Additionally, Retigabine, a neuronal KCNQ2-5(K(v)7.2–7.5) ion channels opener, has not yet been approved for use in pediatric populations. Although it may offer targeted therapy for *KCNQ2* encephalopathy, its application is limited due to its side effects, such as urinary retention and skin and retinal pigmentation [14].

Most pathogenic changes found in *KCNQ2*-EOEE are de novo heterozygous missense mutations or deletions, which cause more serious functional defects in potassium current function [[15], [16], [17], [18], [19], [20], [21]]. Whole exome sequencing identified p. W218C variation in the *KCNQ2* gene in the patient studied. It was determined to be a spontaneous new missense mutation in the S4S5 connection region following Sanger sequencing and qPCR verification. Previous studies have found that this region can affect the voltage sensitivity and the stability of channel openings. The mechanism for this is that PIP2 interacts with the S4S5 junction region to up-regulate the current amplitude and voltage sensitivity of KCNQ2 channels. After channel activation, PIP2 interacts with the S4S5 junction region and participates in channel gating [22,5]. Consequently, PIP2 controls the activity of the voltage sensor and the opening of the channel hole through the S4–S5 connection area. The pathogenic variant reported in this study was highly conserved in many species. PROVEAN and PolyPhen-2 tools predicted the pathogenic variant to be harmful. Further comparative structural analysis of wild-type and mutant KCNQ2 proteins revealed that the pathogenic variant may change the local secondary structure and function of the protein. Based on the findings regarding the structural changes in the interaction between mutant KCNQ2 and PIP2, we hypothesize that pathogenic mutations may contribute to the development of epilepsy in children by weakening the binding of PIP2 to mutant KCNQ2. This alteration results in decreased voltage sensitivity or accelerated inactivation of KCNQ2 channels, ultimately leading to a reduction in neuronal M currents. The inhibition of M current will lead to excessive excitation and synchronous discharge of neurons, causing seizures. Consequently, this pathogenic variant may destroy the function of the S4S5 junction region and seriously impair potassium current. Thus, the patient exhibited a severe clinical phenotype. The T217 mutation can lead to benign familial neonatal seizures in other variations of the previous S4S5 connection region instead of causing early epileptic encephalopathy [23,24]. This is because it only causes a partial loss of potassium current or a comparable current [25,26].

In summary, the pathogenic variants of *KCNQ2* have been observed in neonatal developmental epileptic encephalopathy, refractory epilepsy, and intellectual disability [27]. This study reported a novel *KCNQ2* mutation in the S4S5 junction region, including its genotype and phenotype. This broadens the genotype-phenotype relationship spectrum of variation and provides reference and clues for clinical genetic counseling, early intervention, and subsequent research.

## CRediT authorship contribution statement

**Juhua Yang:** Writing – original draft, Methodology, Investigation, Formal analysis, Data curation. **Yuping Huang:** Visualization, Validation, Resources. **Zhijun Chen:** Validation, Investigation, Data curation. **Jiaheng Peng:** Validation, Investigation. **Kangyu Li:** Investigation. **Lijuan Huang:** Investigation. **Jie Yang:** Writing – review & editing, Supervision, Project administration, Funding acquisition, Conceptualization. **Chunhui Yang:** Writing – review & editing, Validation, Supervision, Data curation, Conceptualization.

## Ethics approval and consent to participate

This study was conducted in accordance with the declaration of Helsinki. All study protocols were approved by the Human Ethics Committee of Zhongshan Boai Hospital (KY-2024-011-02).

## Funding

This work was supported by the 10.13039/501100003453Natural Science Foundation of Guangdong Province (2022A1515010427).

## Declaration of competing interest

The authors declare that they have no known competing financial interests or personal relationships that could have appeared to influence the work reported in this paper.

## Data Availability

Data will be made available on request.
